# Moderate Intensity of Treadmill Exercise Rescues TBI-Induced Ferroptosis, Neurodegeneration, and Cognitive Impairments via Suppressing STING Pathway

**DOI:** 10.1007/s12035-023-03379-8

**Published:** 2023-05-16

**Authors:** Jie Chen, Tong Zhu, Dongyu Yu, Bing Yan, Yuxiang Zhang, Jungong Jin, Zhuojin Yang, Bao Zhang, Xiuli Hao, Zhennan Chen, Chunxia Yan, Jun Yu

**Affiliations:** 1 College of Forensic Medicine, Xi’an Jiaotong University Health Science Center, Xi’an, 710061 Shaanxi China; 2 The Key Laboratory of Forensic Medicine (Xi’an Jiaotong University), National Health Commission of China, Xi’an, 710061 Shaanxi China; 3 Clinical Experimental Center, Xi’an International Medical Center Hospital, Xi’an, 710100 Shaanxi China; 4 Academy of Bio-Evidence Science, The Science and Technology Innovation Port in Western China, Xi’an Jiao Tong University, Xi-Xian New Area, 710115 Shaanxi China

**Keywords:** Traumatic brain injury, Ferroptosis, Treadmill exercise, Stimulator of interferon genes, Neurodegeneration, Cognitive impairments

## Abstract

Traumatic brain injury (TBI) is a universal leading cause of long-term neurological disability and causes a huge burden to an ever-growing population. Moderate intensity of treadmill exercise has been recognized as an efficient intervention to combat TBI-induced motor and cognitive disorders, yet the underlying mechanism is still unclear. Ferroptosis is known to be highly implicated in TBI pathophysiology, and the anti-ferroptosis effects of treadmill exercise have been reported in other neurological diseases except for TBI. In addition to cytokine induction, recent evidence has demonstrated the involvement of the stimulator of interferon genes (STING) pathway in ferroptosis. Therefore, we examined the possibility that treadmill exercise might inhibit TBI-induced ferroptosis via STING pathway. In this study, we first found that a series of ferroptosis-related characteristics, including abnormal iron homeostasis, decreased glutathione peroxidase 4 (Gpx4), and increased lipid peroxidation, were detected at 44 days post TBI, substantiating the involvement of ferroptosis at the chronic stage following TBI. Furthermore, treadmill exercise potently decreased the aforementioned ferroptosis-related changes, suggesting the anti-ferroptosis role of treadmill exercise following TBI. In addition to alleviating neurodegeneration, treadmill exercise effectively reduced anxiety, enhanced spatial memory recovery, and improved social novelty post TBI. Interestingly, STING knockdown also obtained the similar anti-ferroptosis effects after TBI. More importantly, overexpression of STING largely reversed the ferroptosis inactivation caused by treadmill exercise following TBI. To conclude, moderate-intensity treadmill exercise rescues TBI-induced ferroptosis and cognitive deficits at least in part via STING pathway, broadening our understanding of neuroprotective effects induced by treadmill exercise against TBI.

## Introduction

Traumatic brain injury (TBI), one of the leading causes of disability and mortality, is a critical health concern worldwide. It is estimated that more than 10 million TBI cases occur annually worldwide, and at least 50% survivors are often left with permanent cognitive deficits many years after a major head injury. Surprisingly, long-term cognitive impairments, such as memory decline, loss of consciousness, and depression, may even persist in survivors with mild TBI, which strongly affects the quality of life in patients [1]. Despite the intensive research, there is still no effective pharmacological option to treat TBI-induced cognitive deficits. Recently, moderate-intensity physical exercise, a non-pharmacological intervention, has gained increasing attention for its neuroprotective effects. For example, treadmill training lowers tissue sensitivity to glucocorticoids, and hypercorticosteronemia is a significant risk for neurodegenerative diseases caused by TBI. In addition, elevated lactate level induced by exercise training is essential for maintaining long-term potentiation (LTP) of synaptic strength [2]. Moreover, many studies have attributed the neuroprotective effects of physical exercise after TBI to reduced neuroinflammation, increased brain-derived neurotrophic factor (BDNF) expression, inhibition of astrocytic reactions, and promotion of angiogenesis [3]. However, less is known regarding the effects of ferroptosis, a new form of regulated cell death, on neurological function recovery induced by physical exercise following TBI.

Ferroptosis, distinct from other known cell death pathways, is an iron-dependent form of regulated necrosis, which is driven by a redox imbalance between oxidation and antioxidant system. Despite the fact that the term ferroptosis was coined in 2012, the number of published studies on ferroptosis has been growing exponentially over the past few years [4]. Mounting evidence has shown that the role of ferroptosis has been extensively documented in a plethora of pathological diseases, particularly in cancer and ischemia–reperfusion (I/R) injuries [5, 6]. More importantly, growing evidence shows that ferroptosis is highly involved in the pathophysiological process of secondary brain injury, including glutamate excitotoxic injury, mitochondrial dysfunction, and neuronal death, particularly in the acute phase post TBI [7, 8]. For example, TBI led to a significant increase of ferroptosis-related genes at 6 h post TBI. In addition to shrunken mitochondria, the increase of malondialdehyde (MDA) and lipid reactive oxygen species (ROS) was observed at 3 days following TBI [8]. However, the pathological role of ferroptosis in chronic stage after TBI is still poorly understood, necessitating further dissection of the effects of ferroptosis on TBI-induced chronic and persistent cognitive impairment.

Stimulator of interferon genes (STING)-induced neuroinflammatory responses are known to be critical in the deleterious process after TBI [9]. In TBI conditions, DNA leakage from dying cells into the cytoplasm potentially activates cytosolic cyclic GMP-AMP (cGAMP) synthase (cGAS). After recognition of foreign pathogenic or mislocalized DNA, STING is canonically activated by cGAMP, thereby facilitating the production of inflammatory cytokines and chemokines [10]. However, prolonged or chronic neuroinflammation causes a toxic microenvironment deleterious to neuronal cell viability after brain injury, eventually aggravating cognitive and stress-related dysfunction [11]. It is recognized that STING inhibition limits neurodegeneration progression and leads to at least a partial rescue of cognitive abnormalities and cortical degeneration post TBI [12]. For example, TBI induced polarization of microglia toward M1 phenotype, and STING depletion effectively reduced multiple pathological features of M1-polarization and produces an M2-polarizing environment. And reduced lesion volume, attenuated inflammatory response, and improved neurobehaviors were observed in STING^−/−^ mice after brain injury [13]. However, the function of STING in TBI-induced ferroptosis at later time points is still elusive, and whether STING is involved in the anti-ferroptosis effects of treadmill exercise remains poorly understood.

In the current study, we hypothesized that moderate intensity of treadmill exercise could inhibit TBI-induced ferroptosis at least partially via STING pathway at the chronic phase after TBI. To test this hypothesis, we first detected the ferroptosis level at the chronic stage (44 days) after TBI. Subsequently, we investigated whether treadmill exercise or STING knockdown exerts anti-ferroptosis effects and alleviates cognitive impairments. Finally, we explored whether STING overexpression reverses ferroptosis inhibition and ease of neuronal damage induced by treadmill exercise post TBI.

## Materials and Methods

### Animals

Adult male C57BL/6 J mice (6–8 weeks, 25–30 g) were obtained from Xi’an Jiaotong University Animal Laboratory. All mice were randomly housed in a controlled environment (5 animals per cage, 55 ± 5% humidity, 23 ± 1 °C, 12:12-h light/dark cycle) and equilibrated for 7 days before use. Standard laboratory food and water were available ad libitum. All the animal experiments and procedures received approval from Xi’an Jiaotong University Laboratory Animal Administration Committee and were conducted strictly in agreement with Xi’an Jiaotong University Guidelines for Animal Experimentation [14]. Best efforts were made to minimize the suffering of animals and the number of animals used.

### Traumatic Brain Injury

The controlled cortical impact (CCI) injury model of the mouse was established using Pneumatic Impact Device (AMS 201, AmScience). Briefly, mice were anaesthetized with 2% sodium pentobarbital and subsequently fixed on a stereotactic device (RWD Life Science Co, Shenzhen, China). Under sterile conditions, a midsagittal skin incision was conducted on the scalp. A 3.0-mm diameter of circular craniotomy was then performed over the left frontal cortex (center of the craniotomy: 2.0 mm posterior and 2.0 mm lateral to the bregma). The exposed brain cortex was perpendicularly struck by a 2.0-mm-diameter flat tip (depth: 1.0 mm, dwell time: 70 ms, pressure: 10 kPa). After the impact, tissue adhesive (3 M) was used to close the scalp. After about an hour of recovery, the mouse resumed motor activity and was then transported back to their home cages. The mice in sham group received the identical surgical procedures, except for the CCI injury [15].

### Treadmill Exercise Protocol

An animal treadmill (Model JD-PT, Jide Instrument, Shanghai, China) was used. Treadmill exercise training was performed between 09:00 and 10:00 pm [16]. Since mice are more active in darkness, a thick dark paper was covered on the front portion of the treadmill lines. The treadmill training protocol was adapted and developed according to previous studies [17, 18]. Treadmill training consists of the acclimation phase and exercise phase. During the acclimation phase, mice were forced to run at a low-intensity level for three consecutive days (a speed of 2 m/min for the first 5 min, 5 m/min for the next 5 min, and then 8 m/min for the last 20 min, 0° slope) [18]. After this period, the moderate-intensity treadmill exercise phase was carried out for 4 weeks (5 days per week, a speed of 10 m/min for the first 5 min, 12 m/min for the next 5 min, and then 15 m/min for the last 20 min, an inclination of 0°) [17]. The energy consumption of moderate-intensity exercise is 3–6 times higher than that of basic metabolism, and the intensity range is close to 50–55% of the maximum oxygen uptake. A soft brush was used to stimulate the mice to achieve maximal effort during training sessions, and no electronic shocks were employed to decrease the stress effect of running on the treadmill [19]. The sham group mice were left on the switched-off treadmill without running for the same duration.

### Virus Injection

Prior to surgery, each mouse was anesthetized with 2% sodium pentobarbital and mounted on a stereotactic frame (RWD Life Science Co, Shenzhen, China). Eye drops were applied to prevent corneal drying and a heat pad (RWD, Shenzhen, China) was used to maintain the core body temperature of the mice at 37 °C [20]. To knockdown the expression of STING, the rAAV2/9-U6-shRNA(STING1)-CMV-EGFP (2.0 × 10^12^ vg/ml, 5′-CCAACAGCGTCTACGAGA-3′) was used. And rAAV2/9-U6-shRNA(Scramble)-CMV-EGFP (2.0 × 10^12^ vg/ml, 5′-CCTAAGGTTAAGTCGCCCTCG-3′) was used as a negative control. To overexpress the expression of STING, rAAV9-hSyn-STING1-P2A-EGFP (1.0 × 10^12^ vg/ml, NM_001289591.1) was used. And rAAV9-hSyn-EGFP (1.0 × 10^12^ vg/ml) was used as a negative control. The above virus was all purchased from BrainVTA (Wuhan, China). A volume of 350 nl virus was injected into the target coordinate (AP: − 1.5 mm, ML: 2.0 mm, DV: − 1.0 mm) at a speed of 50 nl/min, using the Hamilton syringe (65,460–02, Stoelting, Wood Dale, IL, USA). And the barrier inner diameter of needle is 0.343 mm. After the virus injection, the needle was placed in the target brain region for an additional 10 min to prevent backflow. Twenty-one days later, the mice were subjected to the controlled cortical impact (CCI). The mice of the sham group underwent the same surgical procedures except for the virus injection.

### Experimental Design

**Fig. 1:**
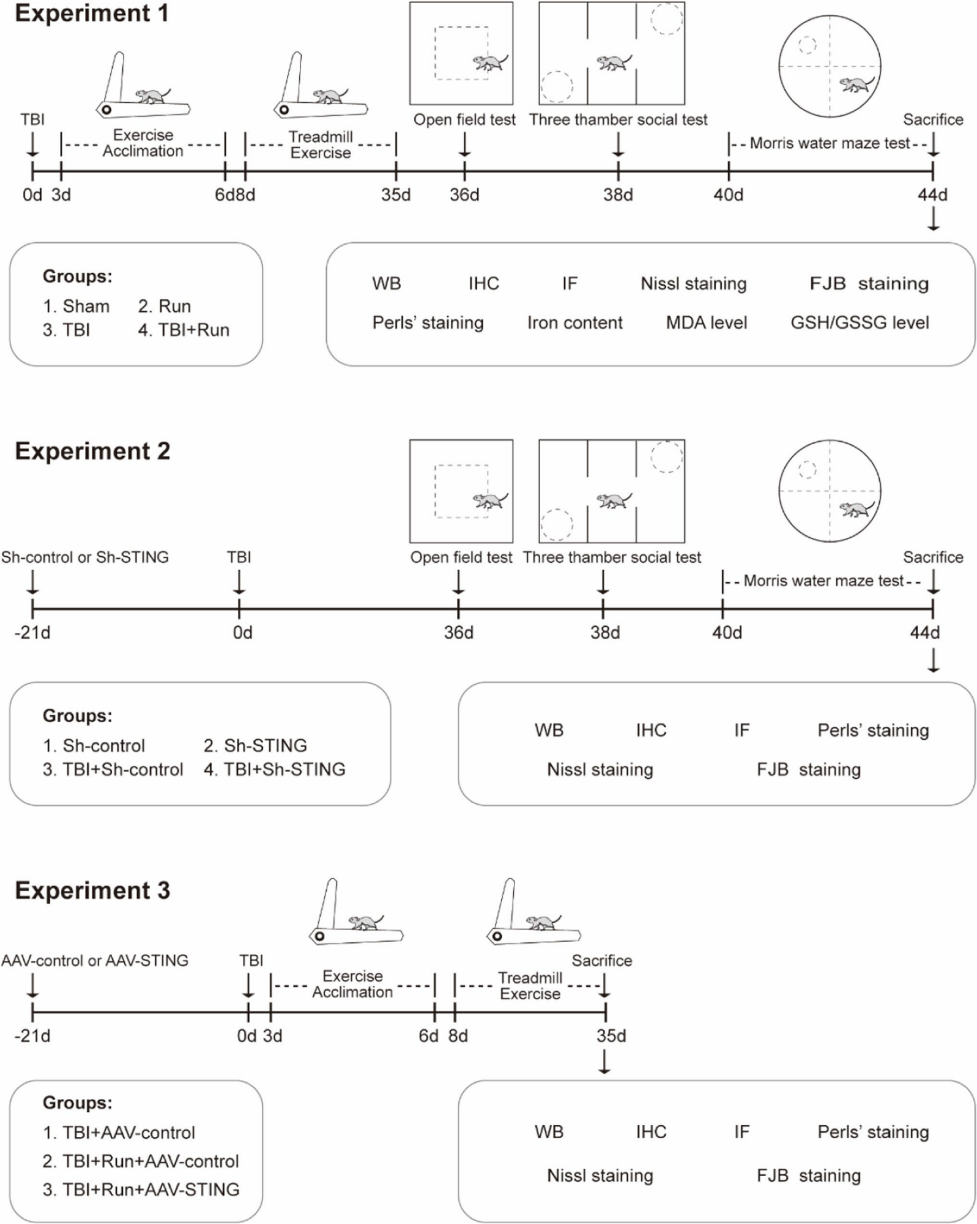
Experimental design and timeline. **A** Experiment 1 was designed to detect the protective role of treadmill exercise against ferroptosis and cognitive deficits caused by TBI. **B** Experiment 2 was designed to explore the effects of STING knockdown on TBI-induced ferroptosis and neurological dysfunction. **C** Experiment 3 was to investigate whether the treadmill exercise inhibits ferroptosis induced by TBI at least partly via STING pathway

This study consists of three experiment parts, and the detailed experimental design was shown in Fig. 1.

#### Experiment 1

To investigate the effects of moderate intensity of treadmill exercise on ferroptosis and cognitive outcome post TBI, the mice were divided into 4 groups (*n* = 20): sham group, run group, TBI group, and TBI + run group. Sham group: the mice received only the same surgical procedures, except for the CCI injury; run group: mice were subjected to treadmill exercise training; TBI group: mice were subjected to CCI injury; TBI + run group: mice were forced to receive the treadmill training from 4 to 35 days after TBI. Then, a series of behavioral tests (open field test (OFT), Morris water maze (MWM), and three-chamber social test (TCST)) were performed at indicated time points. After the mice were sacrificed, western blotting, immunohistochemistry staining, immunofluorescence staining, Perls’ blue staining, Nissl staining, FJB staining, iron content, MDA level, and GSH/GSSG level were conducted, respectively.

#### Experiment 2

To better clarify the role of STING involved in the pathophysiology following TBI, the mice were divided randomly into 4 groups (*n* = 12): Sh-control group, Sh-STING group, TBI + Sh-control group, and TBI + Sh-STING group. Mice assigned to Sh-control and TBI + Sh-control groups received the injection of rAAV2/9-U6-shRNA(Scramble)-CMV-EGFP; mice assigned to Sh-STING and TBI + Sh-STING groups received the virus injection of rAAV2/9-U6-shRNA(STING1)-CMV-EGFP. Twenty-one days later, mice assigned to TBI + Sh-control and TBI + Sh-STING groups were subjected to CCI injury. Subsequently, all mice received a series of behavioral experiments (OFT, MWM, and TCST) at indicated time points. Finally, all mice were sacrificed at 44 days post TBI.

#### Experiment 3

To explore whether treadmill exercise exerts neuroprotective effect via STING pathway after TBI, mice were divided randomly into 3 groups (*n* = 12): TBI + AAV-control group, TBI + run + AAV-control group, and TBI + run + AAV-STING group. In the TBI + AAV-control and TBI + run + AAV-control groups, mice were injected with rAAV2/9-hSyn-EGFP; in the TBI + run + AAV-STING group, mice were injected with rAAV2/9-hSyn-STING1-P2A-EGFP. Twenty-one days later, all mice were subjected to CCI injury. Subsequently, mice assigned to TBI + run + AAV-control and TBI + run + AAV-STING groups were then subjected to treadmill training. Finally, all mice were sacrificed at 35 days post TBI.

### Preparation of Specimens

For western blotting and iron content assay of the injured cortex, we killed mice by using anesthesia administration of 2% sodium pentobarbital and phosphate-buffered saline (PBS) transcardial perfusion. Then, we harvested the injured cortex and preserved them in liquid nitrogen until measure.

To conduct immunohistochemical and immunofluorescence staining, Perls’ Prussian blue staining, Nissl staining, and Fluoro-Jade B (FJB) staining, each mouse was anesthetized intraperitoneally with 2% sodium pentobarbital, and then received PBS transcardial perfusion, followed by 4% paraformaldehyde (PFA). Subsequently, we collected brain tissues and kept them in 4% PFA for 24 h at 4 °C. Subsequently, each brain was dehydrated with 15% and 30% sucrose for 24 h at 4 °C successively. Then, brain samples were embedded in optimal cutting temperature (OCT) and cut into 15-μm coronal sections using a Microtome Cryostat (Leica CM 1850), and preserved at − 80 °C before use.

For evaluation of iron content, MDA, and GSH/GSSG activity of serum, mice were anaesthetized with 2% sodium pentobarbital, and then blood was collected by cardiac puncture, placed for 25 min at room temperature, centrifuged at 3000 rpm for 15 min to obtain serum, and serum was stored at − 80 °C until processed [21, 22].

### Western Blotting

Western blotting for the expression of STING, ferritin heavy chain (Fth), transferrin receptor (Tfr1), ferroportin (Fpn), SLC7A11 solute carrier family 7 member 11 (xCT), Gpx4 (glutathione peroxidase 4), and acyl-CoA synthetase long-chain family member 4 (Acsl4) were conducted, as previously described [23]. The injured ipsilateral cortex (the center: the impact site of the ipsilateral cortex, the range: 2 × 2 × 2 mm) was homogenized in ice-cold RIPA lysis buffer (Beyotime, Jiangsu, China) containing the phenylmethanesulfonyl fluoride (PMSF, Beyotime). After sufficient lysis, the tissue homogenates were centrifuged at 12,000 rpm for 20 min at 4 °C and the supernatants were collected. Protein concentration was quantified using NanoDrop 2000C (Thermo Scientific, USA). The protein extracts (20 µg/sample) were separated on 8% or 10% SDS-PAGE and then electro‐transferred onto polyvinylidene fluoride (PVDF) membranes (Millipore). The PVDFs were blocked in 5% nonfat milk in TBST for 2 h and then incubated with the following primary antibodies overnight at 4 °C: STING (1:1000, 19,851–1-AP, proteintech), Fth (1:1000, CY5648, abways), Tfr1 (1:1000, CY5396, abways), Fpn (1:1000, 26,601–1-AP, proteintech), xCT (1:1000, CY7046, abways), Gpx4 (1:1000, CY7046, abways), Acsl4 (1:1000, CY10198, abways), and β-actin (1:1000, 66,009–1-Ig, proteintech). After three times of washing with TBST, membranes were treated with anti-rabbit secondary antibody (1:1000, SantaCruz) or anti-mouse secondary antibody (1:1000, SantaCruz) for 1 h at room temperature. Band detection was performed using the ECL chemiluminescence system (Clinx Science Instruments, China). Densitometric analysis was conducted, using NIH ImageJ software (Bethesda, MD, USA) [23].

### Immunohistochemical and Immunofluorescence Staining

Brain tissues were subjected to gradient sucrose dehydration and cut into sections of 15 μm, after being fixed with 4% paraformaldehyde for 24 h. Subsequently, brain slices were washed in PBS for 10 min, permeabilized in 0.3% TritonX-100 and 10% goat serum for 1 h under 37 °C. Then, tissue sections were cultivated by the primary antibodies anti-Gpx4 (1:100, CY6959, abways), anti-Fth (1:100, CY5648, abways), anti-4HNE (1:200, ab46545, abcam), or anti-STING (1:200, 19,851–1-AP, proteintech) at 4 °C overnight. After washing with PBS, the sections were incubated with Alexa Fluor 488-goat anti-rabbit (A48282TR, Invitrogen) and 594-goat anti-rabbit (A-11072, Invitrogen) secondary antibodies for 1 h. Eventually, the nuclei were cultured by DAPI (1:1000, Beyotime) for 5 min and the images were collected using a fluorescent microscope (Nikon, DS-Ri2) [24]. The exposed time of antibodies combining the Alexa Flour 488 in all images was 200 ms, and the exposed time of antibodies combining the Alexa Flour 594 in all images was 500 ms. In addition, the exposed time of DAPI in all images was 50 ms [25, 26].

### Perls’ Prussian Blue Staining

Perls’ staining was performed to detect the cellular iron deposition, as described previously [7]. After washing with PBST for three times (5 min per time), the sections were treated with Perls’ solution (5% potassium ferrocyanide/5% hydrochloric acid) for 30 min. Then, the slices were incubated in solution (0.3% hydrogen peroxide in methanol) for 15 min, which aims to block endogenous peroxidase activity. After rising with PBST for 15 min, the sections were cultured in 3,3‐diaminobenzidine (DAB; Vector Laboratories, Burlingame, USA) for 2 min to develop the signals and then were stained with hematoxylin (Sigma‐Aldrich, St Louis, MO, USA) for counterstaining. Three sections per animal were viewed and photographed under a microscope (Nikon TE300; Nikon). Iron-positive cells were quantified by four randomly selected microscopic fields per image at × 100 magnification surrounding the injury area. Cell quantification was performed in an unbiased manner. The number of cells was measured using NIH ImageJ software (Bethesda, MD, USA).

### Nissl Staining

Nissl staining was performed to evaluate the degree of neuronal damage in the injured cortex. After three washes with PBS for 15 min, frozen brain Sects. (15 μm) were incubated in Nissl staining solution (C0117, Beyotime, China) for 5 min at 37 °C [15]. The normal neurons were characterized by a relatively large soma, abundant cytoplasm, and high levels of Nissl body. However, other cell forms such as condensed nuclei, shrunken vacuoles, and decreased Nissl body represent the injured cells. The pictures were captured using a light microscope and Nissl-positive neurons were counted by using the ImageJ software.

### Fluoro-Jade B (FJB) Staining

In order to identify degenerative neurons, Fluoro-Jade B (FJB) staining was performed according to the manufacturer’s specifications. Briefly, coronal brain sections were incubated with 80% ethanol containing 1% sodium hydroxide for 5 min, followed by 70% ethanol for 2 min. After rinsing with distilled water for 5 min, the sections were immersed in 0.06% potassium permanganate solution for 10 min and then 0.0001% FJB working solution (AG325, Millipore, Germany) for 30 min. Subsequently, the tissue sections were dehydrated with xylene. Images were captured using a fluorescence microscope (Nikon, DS-Ri2) [27]. Cell degeneration counts were analyzed by a blinded observer using NIH ImageJ software (Bethesda, MD, USA).

### Cell Counting

Cell quantification was performed in an unbiased manner. Brain sections at − 1.0, − 2.5, and − 4.0 mm from the bregma were obtained and stained [28]. Approximately 4 to 6 images per section were examined and photographed. Positive cells (4HNE, Fth, Gpx4, iron, FJB-positive) were quantified by four randomly selected microscopic fields per image at × 50 magnification, × 100 magnification, × 200 magnification, or × 400 magnification surrounding the injury area. The number of cells was measured using NIH ImageJ software (Bethesda, MD, USA).

### MDA and GSH/GSSG Activity Assay

To determine lipid peroxide levels and anti-oxidative stress capacity after TBI, GSH and malondialdehyde (MDA) content in the serum was measured using GSH and GSSG Assay Kit (S0053, Beyotime, China) and Lipid Peroxidation MDA Assay Kit (S0131S, Beyotime, China). To obtain the blood serum, mice were anaesthetized with 2% sodium pentobarbital, and blood was collected by cardiac puncture, placed for 25 min at room temperature, and then centrifuged at 3000 rpm for 15 min to obtain serum. Subsequently, serum supernatant was collected and then added to a 96-well plate and assay was then performed according to the manufacturer’s instructions [7]. MDA is an intermediate product of lipid peroxidation MDA and its contents in the serum were examined using thiobarbituric acid (TBA) method. The MDA in the serum reacted with TBA to produce an MDA-TBA adduct that can be quantified (OD = 532 nm) colorimetrically. MDA levels were expressed as μmol/l [7]. Since DTNB and GSH react to produce 2-nitro-5-thiobenzoic acid, which is a yellow-colored product, GSH concentration can be detected by the measurement at 412 nm absorbance. Total glutathione (total GS) content consists of GSH content and GSSG content, and the GSH content was calculated as: Total Glutathione − GSSG × 2. GSH content was expressed as μmol/l [29].

### Iron Content Determination

The concentration of ferrous ion (Fe^2+^) in the serum and injured cortex was estimated by the iron assay kit (E-BC-K773-M, Elabscience). In brief, arterial blood was extracted from the left ventricle and collected into labeled plastic tubes. Then, supernatant (serum) was obtained by centrifuging the tubes at 2500 × g for 15 min. Next, the 55 µl serum was added to the 165 µl buffer solution in each tube, and then the mixture was subjected to the treatment of chromogenic solution for 0.5 h. For tissue samples, after washing in ice-cold PBS, 0.1 g fresh ipsilateral cortex was accurately obtained and then mixed with 0.9 ml buffer solution. Afterwards, the homogenate was centrifuged at 10,000 × g for 10 min at 4 °C and the supernatant was collected. Subsequently, chromogenic solution was added to each supernatant and the mixture was incubated for 30 min at 37 °C. Supernatant’s absorbance was measured at 535 nm to determine the ferrous ion (Fe^2+^) level. Fe^2+^ level was expressed as μmol/l or μmol/gprot [30].

### Morris Water Maze Test

To evaluate the spatial memory performance, MWM was performed at 40 days post TBI. The test device consisted of a large round pool (diameter 120 cm, depth 50 cm) filled with opaque water (23 °C ± 1 °C) to a height of 30 cm. The pool was divided into four quadrants with distinct visual cues hung on the wall. An invisible circular platform, 10 cm in diameter and 2 cm beneath the water surface, was located in the northeast quadrant of the pool. During the acquisition phase, each mouse was trained for five consecutive days, with four trials per day. And the trials in each session were separated by a 10-min break. For each trial, mice were individually positioned facing the wall in one of the four quadrants and gently released into the pool. The mice were allowed up to a maximum of 60 s to reach the hidden platform. After arriving at the platform, mice were allowed to stay there for 15 s and then placed in a dry cage until the beginning of the next trial. If mice failed to find it, all of them were guided to the submerged platform and then rested for additional 15 s. On the sixth day, each mouse was laid in the southwest quadrant and then swam freely for 60 s without the hidden platform during the spatial probe test [31]. All trials and data, including frequency of crossing the platform location, latency to reach the platform, swimming speed, and swimming distance, were automatically recorded and analyzed with a video camera connected to a computer equipped with the Ethovision 5.0 tracking software (Noldus, Netherlands).

### Open Field Test

To investigate the anxious behavior and motor activity, OFT was carried out at 36 days after TBI. The OFT device consists of a black square bottom (50 × 50 cm) and a black wall (50 cm), and the apparatus was divided into the central area (25 × 25 cm) and the marginal area. Mice were laid in the corner of the apparatus and habituated to the environment for 1 min. Then, the mouse was allowed freely to explore their surroundings for 5 min. The box was cleaned with a paper towel saturated in 50% ethanol and dried completely after each test session. The motion trail was recorded with an infrared camera fixed over the box. The data, including distance, mean speed, time spent in the center, and the number of entrances into the center, were collected and analyzed using Ethovision 5.0 tracking software (Noldus, Wageningen, Netherlands) [32].

### Three-Chamber Social Test

To evaluate the sociability and social novelty, TCST was performed at 38 days post TBI. The apparatus consisted of a custom-built plexiglass three-chamber box and an overhead video camera. Each chamber was equally divided at 20 (width) × 40 (length) × 20 (height) cm, and the dividing wall had a small square opening (5 cm wide × 5 cm high) to allow free access to each chamber. The custom-made stainless barred cages (diameter of 8.0 cm, height of 20 cm), allowing nose contact between the bars, were laid out in each of the side chamber to enclose an unfamiliar 5-week-old juvenile mice mouse (strange mouse), which aims to reduce the risk of attacking behavior during test period. The time that the test mouse spent facing the stranger mouse within 2 cm of the stainless barred cages was measured [33].

The test consisted of three consecutive 10-min stages. In the first phase, the test mouse was placed in the middle chamber and allowed to explore the entire apparatus freely, aiming to adapt to the novel environment. During the second period, in order to estimate the sociability ability, the stranger mouse 1, which had no prior contact with the test mouse, was enclosed in the cage of the left-side chamber, and the test mouse was allowed to explore for a duration of 10 min. The amount of time spent around each cage (stranger 1 or empty 2) was measured. The sociability index was calculated, which means the numerical time difference between stranger 1 and empty 2 divided by the total time both in stranger 1 and empty 2: (stranger 1 − empty 2)/(stranger 1 + empty 2). After the sociability phase, the social novelty preference test was performed. For this test, another stranger mouse 2 was placed in the stainless cage of the right-side chamber, which had been empty before, and then the test mouse was allowed to explore the two strangers for a period of 10 min. The amount of time spent around each cage (stranger 1 or stranger 2) was measured. The social novelty index was calculated, which means the numerical time difference between stranger 1 and stranger 2 divided by the total time both in stranger 1 and stranger 2: (stranger 2 − stranger 1)/(stranger 1 + stranger 2). Data acquisition and analysis were performed automatically using Ethovision 5.0 tracking software (Noldus, Wageningen, Netherlands) [34].

### Statistical Analysis

All data were expressed as means ± SEM. Statistical analyses were performed using GraphPad Prism 8 Software (San Diego, CA, USA). The difference between two groups was compared using a two-tailed unpaired Student’s *t*-test. Data analysis was determined using one-way ANOVA followed by Turkey’s post hoc test for multiple comparisons. A *p* value less than 0.05 was considered statistically significant [35].

## Results

### Moderate Intensity of Treadmill Exercise Reduces the Ferroptosis Level at Chronic Phase After TBI

**Fig. 2:**
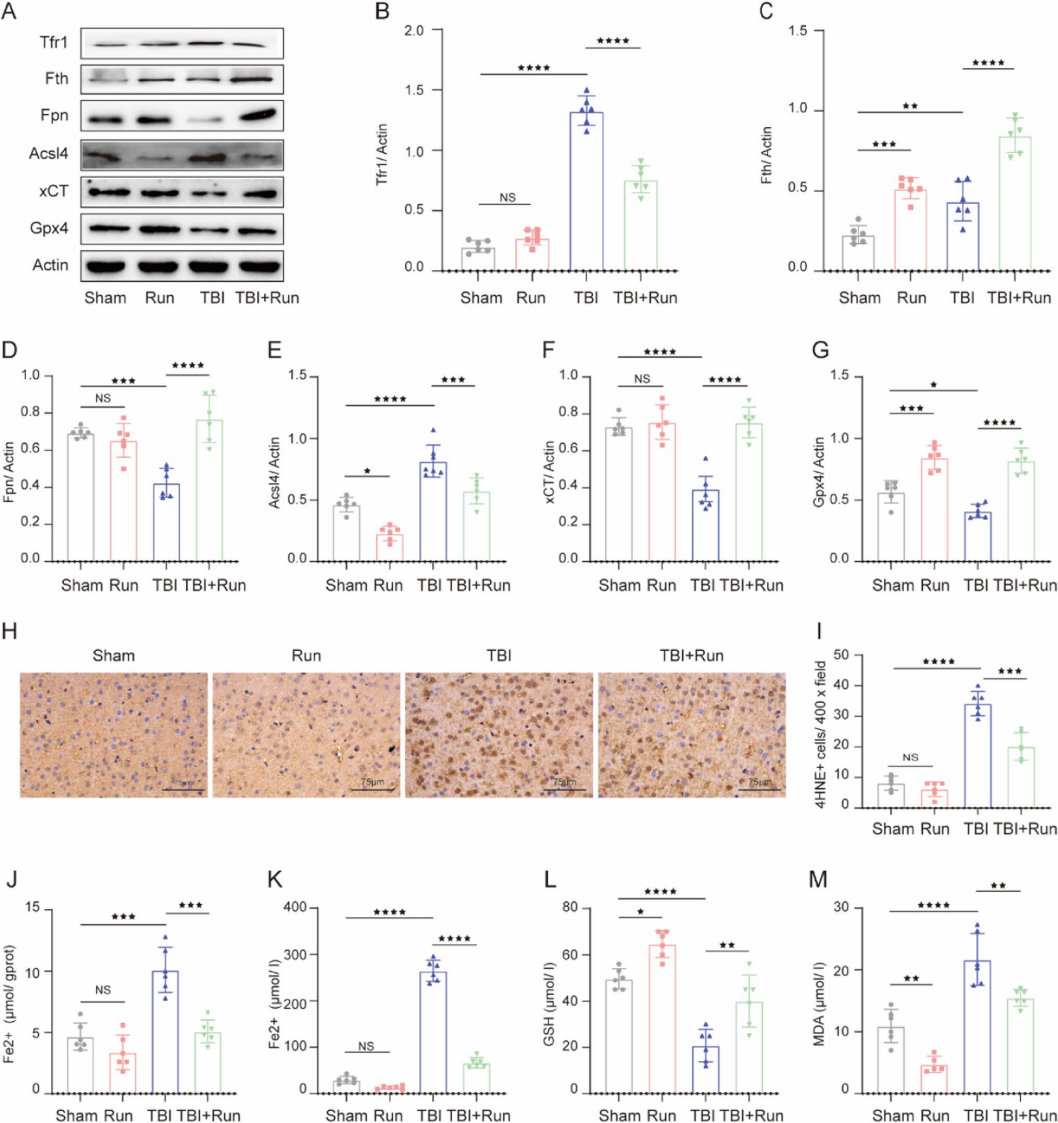
Moderate-intensity treadmill exercise alleviated TBI-induced ferroptosis post TBI. **A** Representative gel bands of Tfr1, Fth, Fpn, Acsl4, xCT, Gpx4, and actin in the injured cortex at 44 days post TBI. **B**–**G** The relative change of Tfr1, Fth, Fpn, Acsl4, xCT, and Gpx4 in each group, and β-actin served as a loading control. **H** Representative immunohistochemical staining images of 4HNE expression in the injured cortex. Scale bar, 75 μm. **I** Quantitative analysis of 4HNE-positive cells in each group. **J** The relative change of Fe^2+^ content in the injured cortex of each group. Fe^2+^ content (**K**), GSSH/GSG level (**L**), and MDA activity (**M**) in the serum of each group. All data are presented as mean ± SEM (*n* = 6) and analyzed using one-way ANOVA followed by Tukey’s post hoc test. For all panels : ⋆*p* < 0.05, ⋆⋆*p* < 0.01, ⋆⋆⋆*p* < 0.001, ⋆⋆⋆⋆*p* < 0.0001 and ns means not statistically significant

To determine whether ferroptosis occurs in the chronic phase after TBI and the effects of moderate intensity of treadmill exercise on TBI-induced ferroptosis, western blotting was performed to detect the expression of ferroptosis-associated proteins, including Tfr1, Fth, Fpn, Acsl4, xCT, and Gpx4 at 44 days after TBI. As shown in Fig. 2A–G, TBI led to a significant increase of Tfr1, Fth, and Acsl4, compared with sham group. On the contrary, we also found decreased expression of Fpn, Acsl4, xCT, and Gpx4 in the damaged cortex after TBI. The above ferroptosis-related protein changes suggested that aberrant iron homeostasis, lipid peroxidation, and impaired antioxidant system were found at the chronic stage after TBI. More importantly, treadmill exercise effectively inversed the above changes induced by TBI, indicating the anti-ferroptosis effects of treadmill exercise after TBI. In addition, immunohistochemical staining results demonstrated that treadmill exercise significantly reduced the upregulated number of 4HNE-positive cells caused by TBI (Fig. 2H and I). We then measured the Fe^2+^ content after TBI, and the results showed that the level of Fe^2+^ was remarkably increased in both the injured cortex and serum following TBI. Notably, treadmill exercise treatment potently reversed this increasing tendency, suggesting that treadmill exercise can alleviate TBI-induced iron overload (Fig. 2J and K). Furthermore, the GSH/GSSG and MDA level in serum was also detected using corresponding assay kits. Interestingly, we found that TBI reduced the level of GSH and increased the level of MDA in serum, and treadmill exercise could significantly invert the above changes (Fig. 2L and M), indicating the antioxidant effects of physical exercise against TBI. To summarize, these results demonstrated that TBI results in a significant increase of ferroptosis at the chronic stage post TBI, and treadmill exercise effectively inhibits TBI-induced ferroptosis.

### Treadmill Exercise Alleviates TBI-Induced Iron Accumulation, Neurodegeneration, and Neuronal Damage Post TBI

**Fig. 3:**
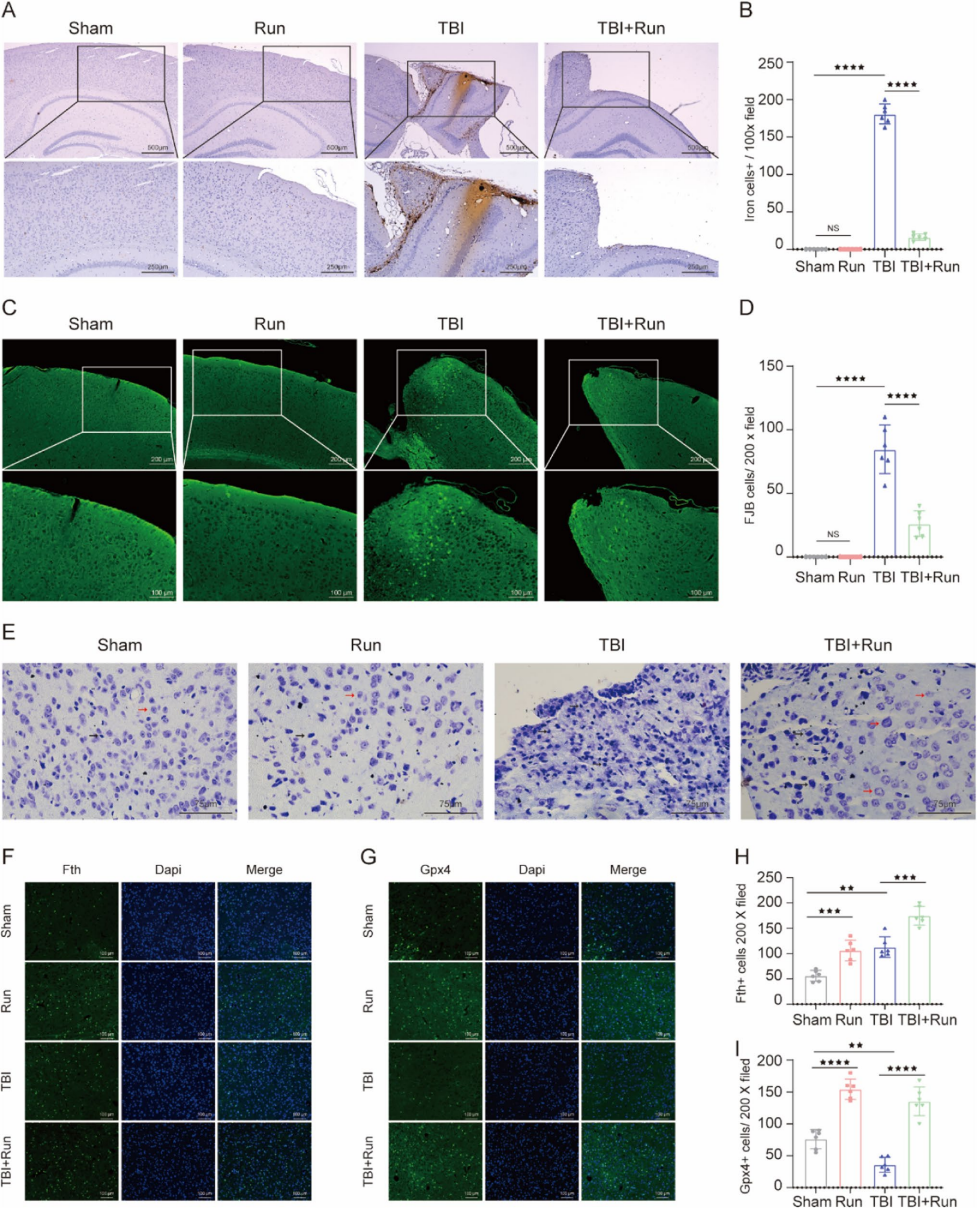
Moderate intensity of treadmill exercise attenuated iron accumulation, neurodegeneration, and neuron damage after TBI. **A** Representative pictures of Perls’ blue-stained cortex sections from sham, run, TBI, and TBI + run groups. Scale bar is 250/500 μm. **B** The number of iron-positive cells per vision field. **C** Representative pictures of Fluoro‐Jade B (FJB) staining in the injured cortex from each group. Scale bar is 100/200 μm. **D** Quantification of FJB-positive cells per vision field. **E** Representative images of Nissl staining from the above group. Black arrows indicate damaged neurons; red arrows indicate intact neurons; scale bar is 75 μm. **F** Representative images of immunofluorescent staining for Gpx4 (green), Fth (green), and DAPI (blue). **G** Representative images of immunofluorescent staining for Fth (green) and DAPI (blue). **H** Quantification of Gpx4-positive cells per vision. **I** Quantitative analysis of Fth-positive cells per vision. All data are presented as mean ± SEM (*n* = 6) and analyzed using one-way ANOVA followed by Tukey’s post hoc test. For all panels: ⋆⋆*p* < 0.01, ⋆⋆⋆*p* < 0.001, ⋆⋆⋆⋆*p* < 0.0001 and ns means not statistically significant

Perls’ blue staining was performed to evaluate the effects of treadmill exercise on TBI-induced iron deposition. We found that the number of iron-positive cells was increased in the TBI group relative to the sham group, whereas the treadmill exercise treatment significantly reduced the number of iron-positive cells, compared to the TBI group (Fig. 3A and B). To determine the effects of treadmill exercise on neurodegeneration level post TBI, FJB staining was performed. We found that TBI led to a remarkable increase in the number of degenerating neurons in the injured cortex. Still, treadmill exercise obviously suppressed the TBI-induced increase of degenerating neurons, compared with TBI group (Fig. 3C and D). To evaluate the changes in neuronal cell outline post TBI, brain sections were stained with Nissl staining. The normal neurons in sham and run groups were characterized by round shape, lilac-blue, large soma, and high levels of Nissl body. However, the injured neurons caused by TBI showed other cell forms such as dark color, condensed nuclei, shrunken vacuoles, and decreased Nissl body. Importantly, we found that the number of injured neurons was obviously decreased in the TBI + run group, compared with TBI group (Fig. 3E). In agreement with our western blotting results, immunofluorescence staining results showed that the expression of Fth was observed to increase in the TBI group compared to sham group, and treadmill exercise effectively increased the TBI-induced overexpression of Fth relative to TBI group (Fig. 3F and H). In addition, we also found that treadmill exercise resulted in a remarkable increase of GPX4 expression, compared with TBI group (Fig. 3G and I). To sum up, these data demonstrated that moderate intensity of treadmill exercise plays a significant role in protecting against TBI-induced iron deposition, neuron injury, and neurodegeneration.

### Treadmill Exercise Attenuates Cognitive Dysfunctions Induced by TBI at Chronic Phase

**Fig. 4:**
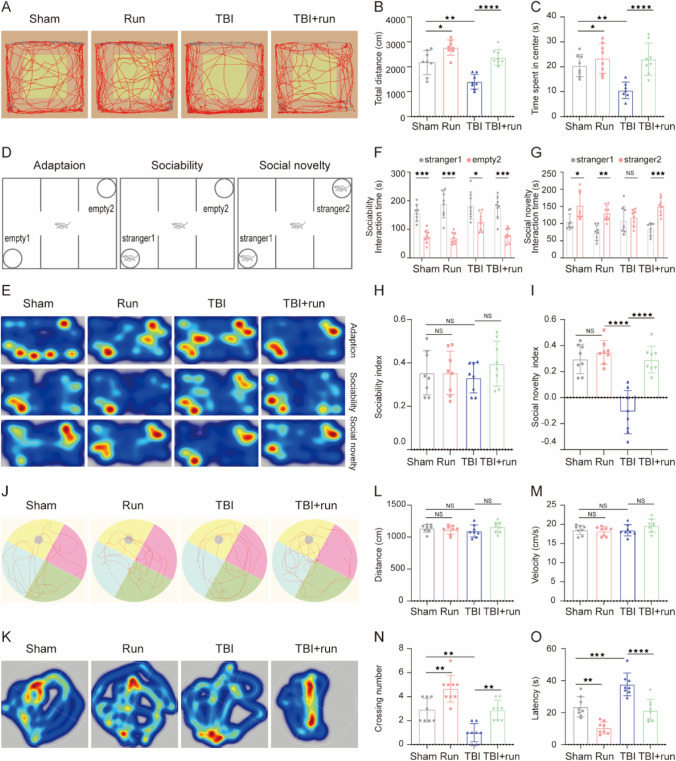
Moderate intensity of treadmill exercise alleviated learning and memory impairments, reduced anxiety, and increased the social novelty post TBI. **A**–**C** The effect of treadmill exercise on anxiety-like behaviors post TBI. **A** Representative tracking of a mouse from sham, run, TBI, and TBI + run groups under OFT. **B** Total distance traveled in the OFT. **C** Time spent in the central field by mice for 5 min. **D**–**I** The effect of treadmill exercise on sociability and social novelty of mouse following TBI. **D** Schematic diagram of the TCST, explaining the procedure of three successive 10-min sessions. **E** Representative heat maps of time spent exploring the three chambers in the social behavior test. **F** During the sociability phase, the interaction time with empty 2 and stranger 1 of mice from each group. **G** During the social novelty phase, the interaction time with stranger 1 and stranger 2 of mice from each group. **H** During the sociability session, the sociability index was calculated by the formula: (stranger 1 − empty 2)/(stranger 1 + empty 2). **I** During the social novelty session, the social novelty index was calculated by the formula: (stranger 2 − stranger 2)/(stranger 1 + stranger 2). **J**–**O** The effect of treadmill exercise on the learning and memory function of mice from different groups. Representative swimming path (**J**) and heatmap images (**K**) of mice from all groups in the MWM test. The traveled distance (**L**) and swimming velocity (**M**) during the probe trail. **N** The number of crossings over the platform position during the probe trail. **O** Escape latency to find the platform during the probe trail. All data are presented as mean ± SEM (*n* = 8) and analyzed using one-way ANOVA followed by Tukey’s post hoc test. For all panels: ⋆*p* < 0.05, ⋆⋆*p* < 0.01, ⋆⋆⋆*p* < 0.001, ⋆⋆⋆⋆*p* < 0.0001 and ns means not statistically significant

To exploit the effects of treadmill exercise on long-term cognitive function, OFT, MWM, and TCST were performed at indicated time points after TBI. In order to evaluate the anxiety level, OFT was performed at 36 days after TBI. Compared with sham group, mice in TBI group traveled shorter total distance and spend less time in center zone. Otherwise, treadmill exercise significantly reversed the abovementioned parameters caused by TBI (Fig. 4A–C). Then, TCST was conducted to detect sociability and social novelty at 38 days following TBI. Sociability was defined as the preference for interacting with the novel mouse compared with the object, and no significant difference in sociability was found among all groups, which means that the sociability was not affected by TBI or treadmill exercise intervention. Social novelty was defined as a preference for a novel mouse over a familiar mouse. Indeed, TBI group mice exhibited an impairment in social novelty, compared to sham group (Fig. 4D–H), whereas treadmill exercise potently inverted TBI-induced decrease of social novelty (Fig. 4I). To evaluate the learning and memory ability, MWM was performed from 40 to 44 days post TBI. There was no difference in distance and velocity in all groups, indicating that the swimming ability was not affected by TBI or treadmill exercise treatment (Fig. 4L and M). In addition, we observed that TBI group showed a significant decrease in platform-crossing number and a longer escape latency to reach the platform compared with sham group, indicating the impaired learning and memory function post TBI. However, the above changes could be reversed by treadmill exercise intervention (Fig. 4J, K, N, and O). Taken together, mice in TBI group showed augmented anxiety-like behavior, impaired social novelty, and spatial learning and memory deficits, whereas treadmill exercise treatment significantly attenuated the above persistent cognitive deficits induced by TBI.

### Sh-STING Reduces Ferroptosis Induced by TBI

**Fig. 5:**
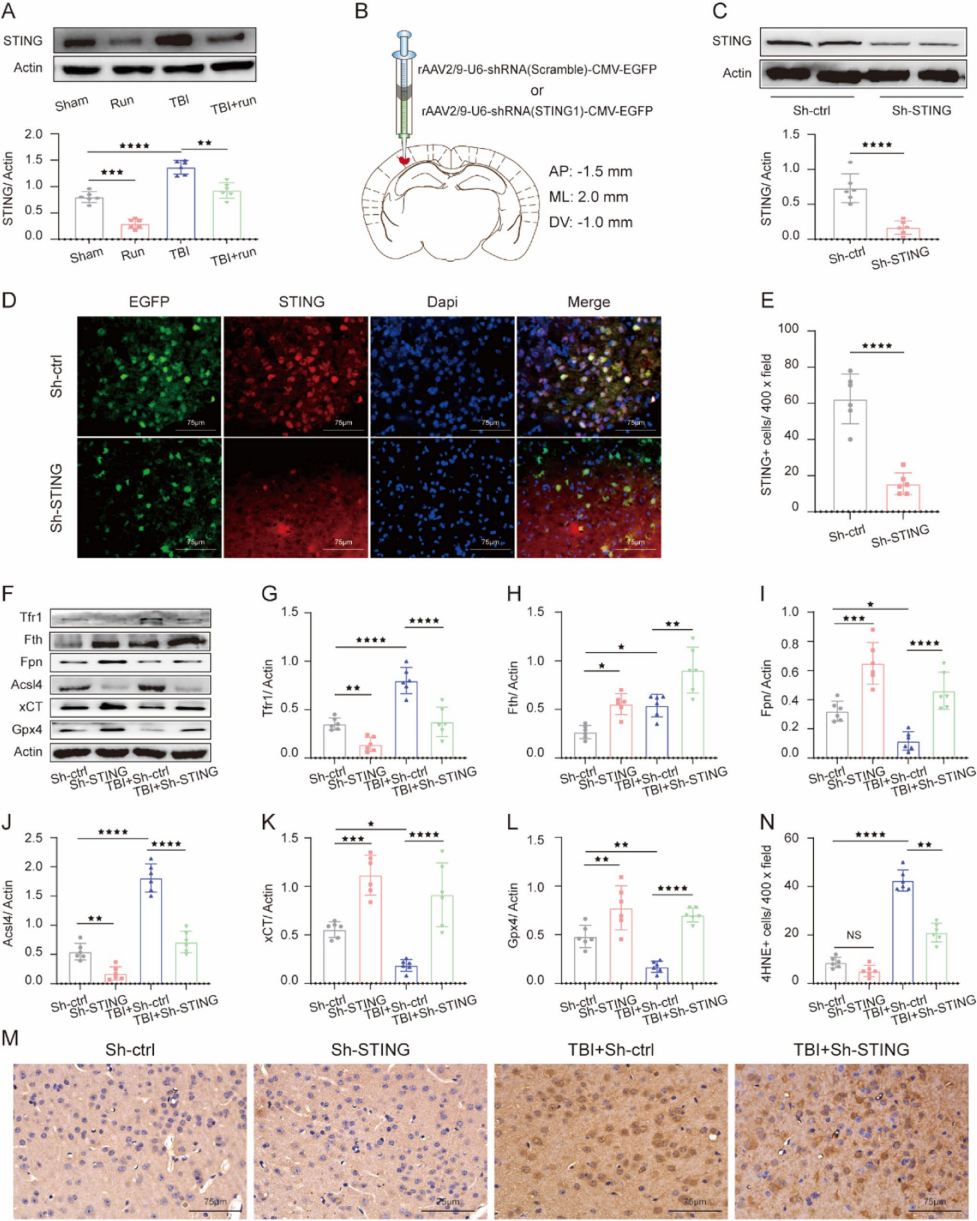
Inactivation of STING pathway suppressed TBI-induced ferroptosis after TBI. **A** Representative immunoblot and quantification of STING in cortex tissue obtained from sham, run, TBI, and TBI + run groups. β-Actin was used as a loading control. **B** Scheme for specific virus infection of Sh-STING and Sh-control. The target site is AP: − 1.5 mm, ML: 2.0 mm, DV: − 1.0 mm. **C** Immunoblot analysis of STING in the cortex after injection of Sh-control or Sh-STING virus. β-Actin was used as a loading control. **D** Representative images of immunofluorescent staining for EGFP (green), STING (red), and DAPI (blue) in the cortex from Sh-control and Sh-STING groups. **E** Quantification of STING-positive cells. Scale bar is 75 μm. **F** Representative western blotting bands of Tfr1, Fth, Fpn, Acsl4, xCT, Gpx4, and actin in the cortex from Sh-control, Sh-STING, TBI + Sh-control, and TBI + Sh-STING groups. **G**–**L** The relative change of Tfr1, Fth, Fpn, Acsl4, xCT, and Gpx4 in the ipsilateral cortex from each group. **M** Representative images of coronal sections demonstrating the distribution of 4HNE immunoreactivity. **N** Quantitative analysis of 4HNE-positive cells from different groups. Scale bar is 75 μm. All data are presented as mean ± SEM (*n* = 6) and analyzed using one-way ANOVA followed by Tukey’s post hoc test. For all panels: ⋆*p* < 0.05, ⋆⋆*p* < 0.01, ⋆⋆⋆*p* < 0.001, ⋆⋆⋆⋆*p* < 0.0001, and ns means not statistically significant

In order to investigate the role of STING in the pathological process of TBI, we examined the expression of STING after TBI. Western botting results revealed that TBI resulted in a remarkable upregulation of STING, compared with sham group. However, treadmill exercise effectively inversed the TBI-induced increasing tendency of STING, indicating the inhibitory effects of physical exercise on STING expression after TBI (Fig. 5A). To determine the impact of STING on ferroptosis following TBI, we conducted the virus injection of Sh-STING to knockdown the STING level in the cortex. And western blotting and immunofluorescence staining results showed that Sh-STING led to a significant decrease of STING, verifying the high efficiency of Sh-STING (Fig. 5B–E). Subsequently, western blotting for ferroptosis-associated biomarkers was measured. Compared with TBI + Sh-control group, the expression of Fth, Fpn, xCT, and Gpx4 was significantly increased in the presence of Sh-STING treatment. In addition, Sh-STING intervention remarkably reduced the increased level of Tfr1 and Acsl4 induced by TBI, which indicated that the impaired iron metabolism, excessive lipid peroxides, and deficits in the antioxidant system were partially restored by Sh-STING treatment (Fig. 5F–L). Moreover, Sh-STING remarkably reduced the number of 4-HNE-positive cells, suggesting the suppression of lipid peroxidation induced by Sh-STING (Fig. 5M and N). In short, these results demonstrated that STING inactivation could effectively attenuate TBI-induced ferroptosis.

### Inactivation of STING Decreased Iron Deposition and Neurodegeneration

**Fig. 6:**
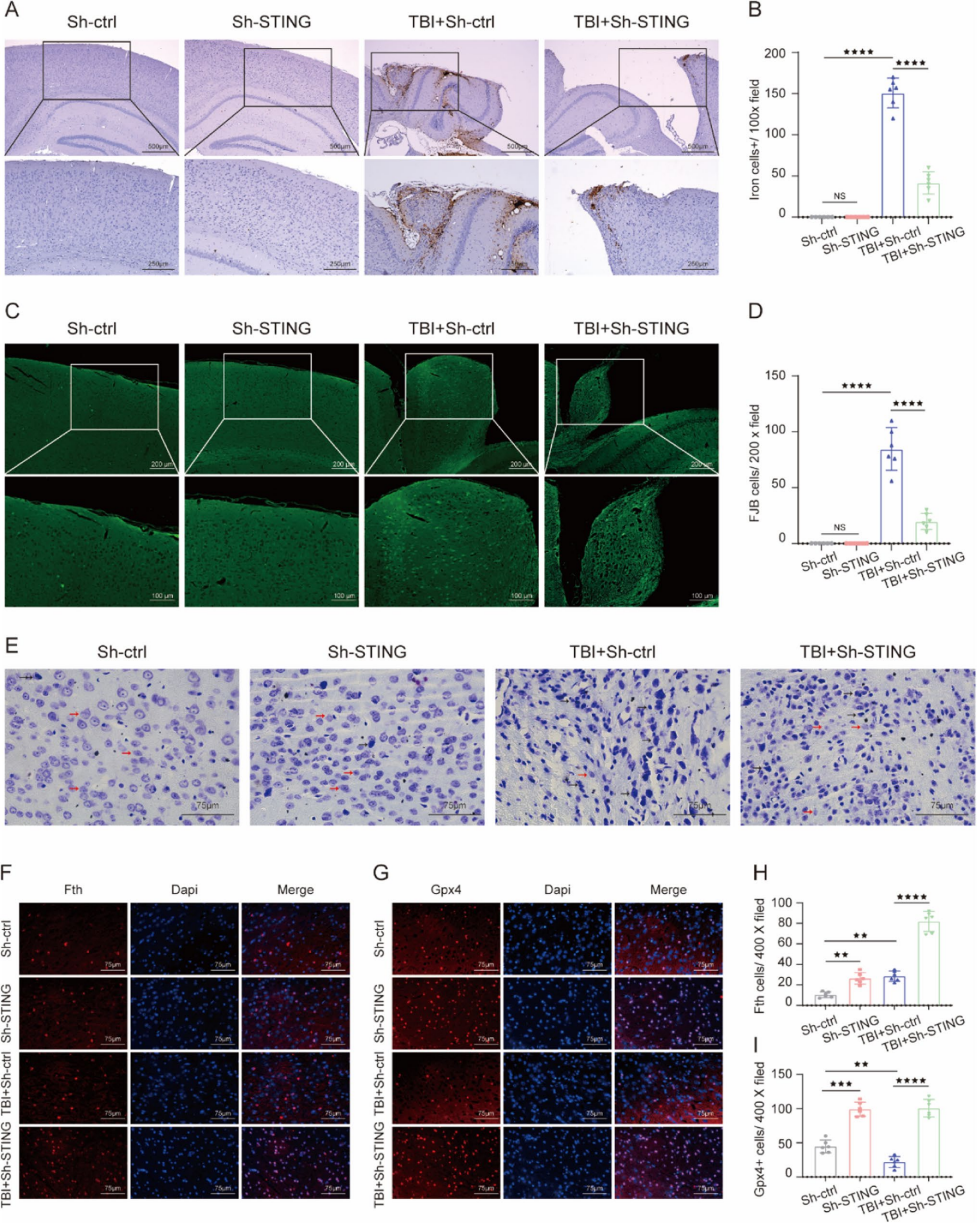
Sh-STING effectively inhibited iron deposition, neurodegeneration, and neuron damage after TBI. **A** Representative images showing the results of iron deposition in injured cortex from Sh-control, Sh-STING, TBI + Sh-control, and TBI + Sh-STING groups. Scale bar is 250/500 μm. **B** Quantitative analysis of iron‐positive cells in each group. Scale bar is 250 μm. **C** Representative images of FJB-stained cortex sections from the above group. Scale bar is 100/200 μm. **D** The number of FJB + cells per vision field. Scale bar is 100 μm. **E** Representative Nissl staining sections of the injured cortex from each group. Black arrows indicate damaged neurons; red arrows indicate intact neurons. Scale bar is 75 μm. **F** Representative IF staining to indicate Fth-positive cells (red) with nuclei fluorescently labeled with DAPI (blue). **G** Representative IF staining to indicate Gpx4-positive cells (red) with nuclei fluorescently labeled with DAPI (blue). **H** Quantification of Gpx4-positive cells per vision field. Scale bar is 75 μm. **I** Quantitative analysis of Fth-positive cells. Scale bar is 75 μm. All data are presented as mean ± SEM (*n* = 6) and analyzed using one-way ANOVA followed by Tukey’s post hoc test. For all panels: ⋆⋆*p* < 0.01, ⋆⋆⋆*p* < 0.001, ⋆⋆⋆⋆*p* < 0.0001, and ns means not statistically significant

To evaluate the effects of Sh-STING on iron deposition post TBI, Perls’ blue staining was conducted. We found that the number of iron-positive cells was markedly reduced in TBI + Sh-STING group, compared with TBI group (Fig. 6A and B). In addition, Sh-STING significantly decreased the TBI-induced increase in the number of degenerating neurons (Fig. 6C and D). Moreover, relative to TBI group, the damaged neuron cells in TBI + Sh-STING group were remarkably increased, indicating that the severity of neuron damage was significantly alleviated by Sh-STING intervention (Fig. 6E). Through immunoblotting, we observed that Sh-STING increased the expression of Fth and Gpx4 following TBI (Fig. 6F–I). To conclude, these observations suggested that STING activation contributes to ferroptosis-related brain damage following TBI.

### Sh-STING Ameliorates TBI-Induced Motor and Cognitive Impairments

**Fig. 7:**
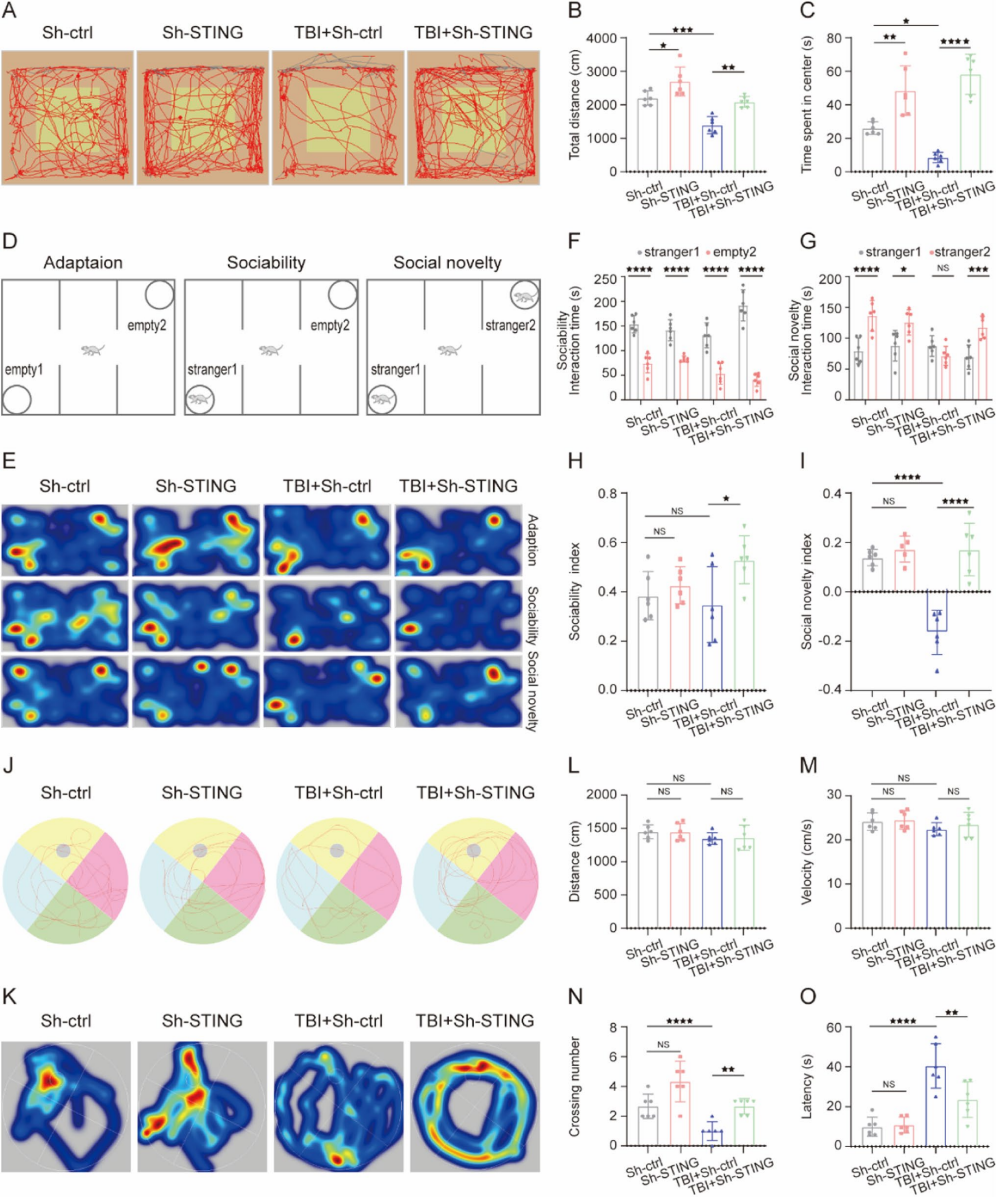
Downexpression of STING reduced persistent cognitive deficits induced by TBI in mice. **A**–**C** The effect of Sh-STING on open field behaviors following TBI. **A** Representative tracks of the open field activity in a mouse from Sh-control, Sh-STING, TBI + Sh-control, and TBI + Sh-STING groups. **B** Total distance traveled moved in the OFT for 5 min. **C** Time spent in the central field by mice for 5 min. **D**–**I** The effect of Sh-STING on sociability and social novelty of mouse following TBI. **D** The schematic diagram of the TCST, explaining the procedure of three successive 10-min sessions. **E** Representative heat maps of time spent exploring the three chambers in the social behavior test. **F** During the sociability phase, the interaction time with empty 2 and stranger 1 of the mouse from each group. **G** During the social novelty phase, the interaction time with stranger 1 and stranger 2 of the mouse from each group. **H** During the sociability session, the sociability index of mouse in each group. **I** During the social novelty session, the social novelty index of the mouse in each group. **J**–**O** The effect of Sh-STING on the learning and memory ability of mice obtained from all groups. Representative swimming pace (**J**) and heatmap images (**K**) of mice from different groups in the MWM test. **L**–**M** The traveled distance and swimming speed in the probe trail. **N** Latency to find the platform in the probe trail. **O** The number of targets crossing in the probe trail. All data are presented as mean ± SEM (*n* = 6) and analyzed using one-way ANOVA followed by Tukey’s post hoc test. For all panels: ⋆*p* < 0.05, ⋆⋆*p* < 0.01, ⋆⋆⋆*p* < 0.001, ⋆⋆⋆⋆*p* < 0.0001 and ns means not statistically significant

The OFT was performed to detect the motor function and anxiety behavior at 36 days following TBI. Interestingly, Sh-STING significantly inverted the decrease in total distance and time in the center zone caused by TBI, suggesting that Sh-STING remarkably attenuated the deficiency in motor function and anxiety level after TBI (Fig. 7A–C). To evaluate the social interaction capacity, TCST was conducted at 38 days after TBI. Compared with the TBI + Sh-control group, Sh-STING remarkably reversed the TBI-induced decrease of social novelty, indicating that social novelty deficits were partly attenuated by the treatment of Sh-STING (Fig. 7D–I). Subsequently, the MWM test was performed to assess the learning and memory ability from 40 to 44 days post TBI. There was no apparent difference in the total distance and velocity among all groups, indicating that Sh-STING does not affect the swimming motor capacity of mice both in basal and TBI conditions (Fig. 7L and M). Compared with TBI + Sh-control group, mice in TBI + Sh-STING group showed a shorter latency to find the platform and crossed the submerged platform more times (Fig. 7J, K, N, and O). To summarize, in addition to enhancing learning and memory and reducing anxiety, inhibition of STING obviously increases social novelty after TBI.

### AAV-STING Reversed the Neuroprotective Effects of Treadmill Exercise Post TBI

**Fig. 8:**
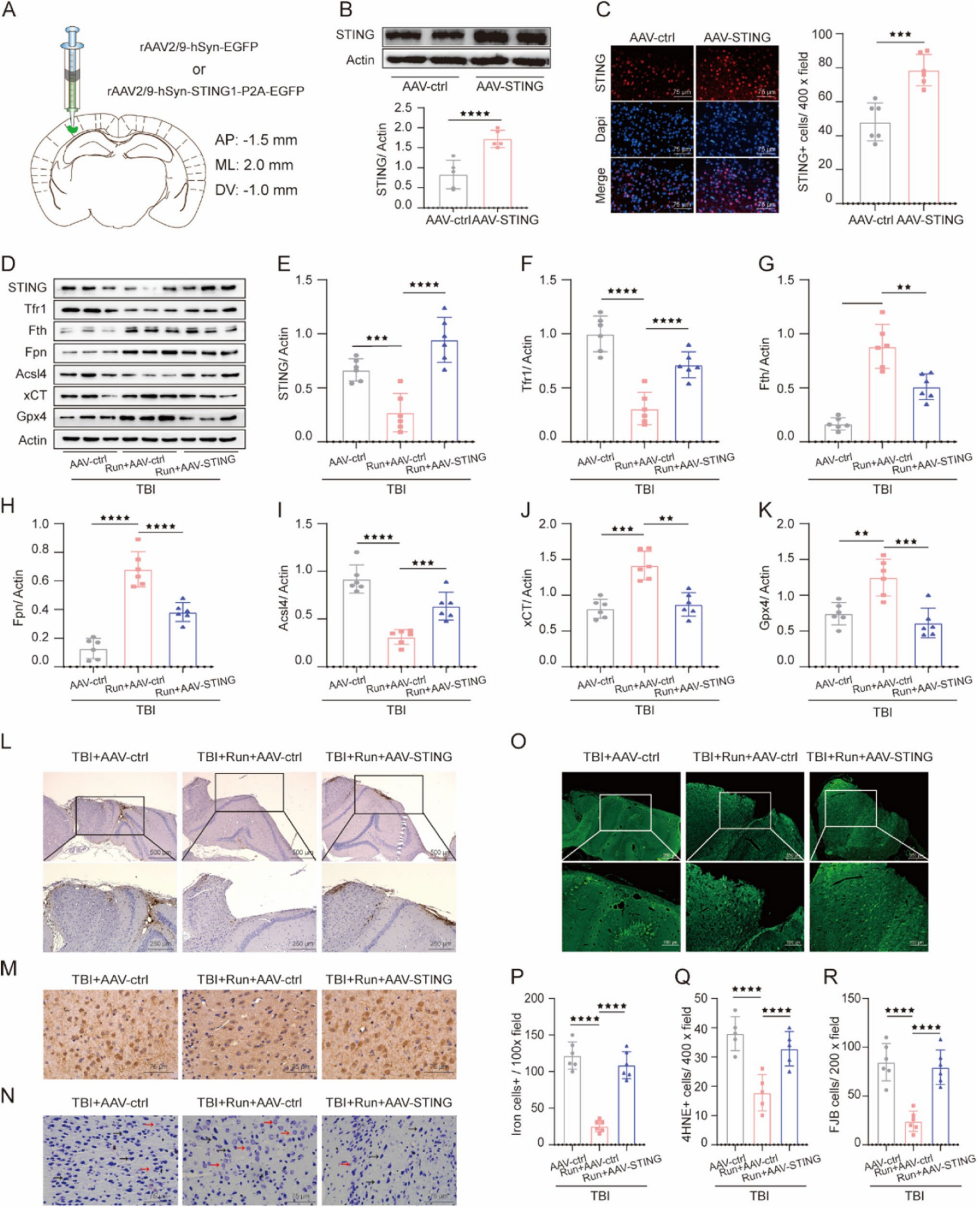
Overexpression of STING largely inversed the inhibition of ferroptosis and alleviation of neurodegeneration induced by treadmill exercise post TBI. **A** Scheme for specific virus infection of AAV-control or AAV-STING. The target site is AP: − 1.5 mm, ML: 2.0 mm, DV: − 1.0 mm. **B** Immunoblot analysis of STING in the cortex after injection of AAV-control or AAV-STING. β-Actin was used as a loading control. **C** Immunofluorescent staining of STING (red) and DAPI (blue) in the cerebral cortex from AAV-control and AAV-STING groups. Scale bar is 75 μm. **D** Representative gel bands of Tfr1, Fth, Fpn, Acsl4, xCT, Gpx4, and actin in the injured cortex from the above groups. **E**–**K** The relative change of Tfr1, Fth, Fpn, Acsl4, xCT, and Gpx4 in each group, and β-actin served as a loading control. **L** Representative images of cortex sections stained with Perls’ Prussian blue in each group. Scale bar is 250/500 μm. **M** Representative views of 4HNE immunostaining in the ipsilateral cortex from each group. Scale bar is 75 μm. **N** Representative images of Nissl staining in the injured cortex from each group. Black arrows indicate damaged neurons. Red arrows indicate intact neurons. Scale bar is 75 μm. **O** Representative pictures of cortex sections stained with FJB. Scale bar is 100/200 μm. **P** Quantification of iron‐positive cells. Scale bar is 250 μm. **Q** Quantification of 4HNE-positive cells per vision field. **R** Quantification of FJB-stained cells per vision field. Scale bar is 100 μm. All data are presented as mean ± SEM (*n* = 6) and analyzed using one-way ANOVA followed by Tukey’s post hoc test. For all panels: ⋆⋆*p* < 0.01, ⋆⋆⋆*p* < 0.001, and.⋆⋆⋆⋆*p* < 0.0001

To ascertain whether the STING pathway was involved in the anti-ferroptosis and neuroprotective effects of treadmill exercise after TBI, we determined to investigate the effects of STING overexpression on TBI-induced ferroptosis and neuron damage. Western blotting and immunoblotting results revealed that AAV-STING led to a remarkable upregulation of STING in the cortex, confirming the high efficiency of AAV-STING (Fig. 8A–C). Notably, overexpression of STING significantly inverted the changes of STING, Tfr1, Fth, Fpn, Acsl4, xCT, and Gpx4 caused by TBI + run + AAV-control, indicating that treadmill exercise plays a significant role in inhibiting ferroptosis at least partly via STING pathway (Fig. 8D–K). In addition, the number of iron-positive (Fig. 8L–P), degenerating (Fig. 8O–R), and damaged cells (Fig. 8N) was markedly increased in the treatment of AAV-STING, compared with TBI + run + AAV-control group. Furthermore, AAV-STING potently increased the expression of 4HNE, indicating that STING activation contributes to the TBI-induced lipid peroxidation (Fig. 8M). Overall, these results provide sufficient evidence to support that moderate intensity of treadmill exercise negatively regulates ferroptosis at least partially by inhibiting STING pathway.

## Discussion

**Fig. 9:**
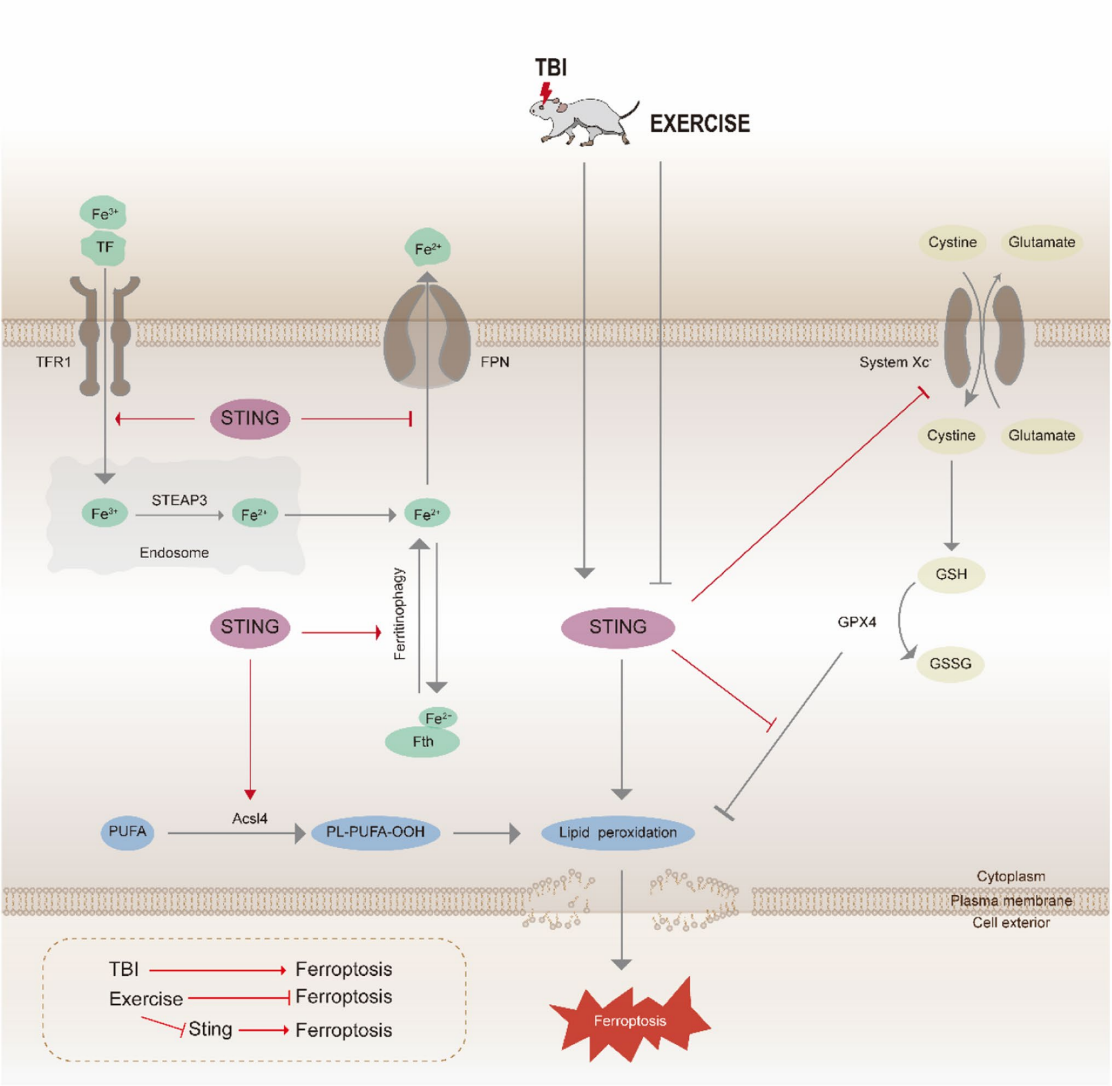
The schematic representation indicating the role of STING in anti-ferroptosis effects induced by moderate intensity of treadmill exercise after TBI

The major findings of our study are as follows: (1) TBI leads to the occurrence of ferroptosis at the chronic phase of TBI. (2) Moderate intensity of treadmill exercise effectively alleviates the iron deposition, decreases the accumulation of lipid peroxides, strengthens the antioxidant system, and attenuates the neuron degeneration level, at least partly via suppressing the STING pathway. (3) Besides improving learning and spatial memory and alleviating anxiety-like behaviors, treadmill exercise results in preponderant enhancement in the social novelty preference after TBI. Taken together, we demonstrated that moderate intensity of treadmill exercise negatively regulates ferroptosis partially via STING pathway within the mouse model of TBI, as shown schematically in Fig. 9.

The pathological process of TBI includes both primary and secondary impairments. Primary injury refers to fractures of the head, tissue displacement, dysfunction of the blood vessels, and intracranial hemorrhage during the initial impact. Due to the extreme limitations of therapeutic approaches against primary injury, more researchers have focused on the pathophysiological changes of secondary injury, which provides more possibility for clinical intervention. Secondary injury is multifaceted and lasting, which is characterized by a complex cascade of events, including glutamate-induced excitotoxicity, mitochondrial damage, abnormal ion metabolism, neuroinflammation, and various forms of cell death [36]. Apart from the classical apoptosis, autophagy, and necrosis, emerging evidence showed that ferroptosis is highly involved in the pathological process of secondary injury post TBI and blocking ferroptosis-related cell death might be a novel way to alleviate the neurobehavioral deficits and promote cognitive recovery [37]. Xie et al. reported that iron deposition, abnormal iron metabolism, and the overexpression of ferroptosis-related genes were detected in the injured cortex at 3 days after TBI, and the administration of ferrostatin‐1 (Fer-1), a specific inhibitor of ferroptosis, significantly reversed the above neuropathological changes [8]. In addition, Rui et al. found that the upregulation of ferroptosis-related molecules and lipid peroxidation was induced at 1 day following TBI, and melatonin treatment exerts neuroprotection effects and reduces memory impairments via suppressing neuronal ferritin H-mediated ferroptosis. Compared with previous studies [7, 15, 38], which primarily focused on the role and mechanisms of ferroptosis in the acute phase post TBI, the innovative finding of our research is that series of ferroptosis-related pathological and morphological characteristics were also detected in the chronic phase (44 days after TBI), which broadens our understanding of the role of ferroptosis during the pathogenesis of TBI and provides compelling evidence of the relationship between ferroptosis and long-term cognitive and mood disorders.

According to the clinical standards of TBI treatment, patients are recommended to proceed with physician-prescribed rest, aiming to prevent repeated injury and reduce metabolic stress during a vulnerable period of post-injury function recovery. However, growing evidence shows that the beneficial outcome of physical rest is minimal, and TBI-induced motor dysfunction might be exacerbated by restricted physical activity and eased through appropriate aerobic exercise. Furthermore, it is a consensus that physical exercise may be one of the best interventions to ameliorate motor impairments and speed cognitive recovery [17]. Due to the highly heterogeneous nature of brain injuries, such as severity and impact regions, there was no clear standard regarding initiation timing, exercise intensity, and exercise type (voluntary vs forced), which are all critical elements for improving function recovery [39]. Initiation of aerobic exercise too early (24 h or 48 h post TBI) might aggravate TBI-induced motor deficits [40]. Furthermore, Karelina et al. reported that exercise onset initiated at 3 days after TBI effectively promotes neuroprotection and ameliorates neurological dysfunction, indicating that 3 days following TBI might be a promising exercise intervention time point for better outcomes [17]. As for the exercise intensity, Shen et al. demonstrated that high-intensity exercise exacerbates physiological and psychological deficits after TBI, highlighting the urgent need to titrate exercise intensity carefully [41]. In addition, it has been documented that mice in the moderate-intensity exercise group showed a better improvement in spatial memory compared to the low-intensity group, suggesting that moderate-intensity exercise may be a more effective approach [42]. Concerning the exercise type, even if voluntary physical exercise leads to fewer stress responses, the forced treadmill is the preferred experimental scheme owing to its more accurate control over exercise intensity and time [43, 44]. Therefore, based on those studies, we designed that mice were subjected to moderate-intensity treadmill exercise at 3 days after TBI in this research. In agreement with previous research [17], our results showed that moderate-intensity exercise reduces neurological deficits and exerts potential neuroprotective effects in TBI models.

Physical activity is recommended not only in healthy individuals but also in people with neurological diseases, such as Alzheimer’s disease and cerebral ischemia–reperfusion (I/R). Notably, the beneficial effects of treadmill exercise go beyond muscle function and involve a cascade of changes in the brain, including angiogenesis, synaptogenesis, and overexpression of neurotrophic factors [45]. For example, Zhao et al. demonstrated that exercise is beneficial for reshaping the structure of related brain regions after ischemic stroke, and the increasing number of dendritic spines and enhancement of synaptic plasticity may be its physiological basis [3]. Treadmill exercise also plays an essential role in protecting mitochondrial bioenergy potential by inhibiting endogenous mitochondrial apoptosis against cerebral ischemia [46]. More importantly, physical activity has been reported to reduce lipid peroxidation and increase the expression of proteins associated with ferroptosis, including nuclear transcription factor E2-related factor 2 (Nrf2) and glutathione peroxidase 4 (Gpx4). Significantly, the administration of erastin (a ferroptosis activator) can reverse the neuroprotective effects of treadmill training, indicating that inhibition of ferroptosis may be a vital pathogenetic process induced by treadmill exercise after cerebral ischemia [47]. However, whether treadmill exercise exerts anti-ferroptosis effects after TBI has not been settled, and if so, what the underlying mechanism is. Interestingly, this is the first report to reveal that moderate treadmill exercise can inhibit TBI-induced ferroptosis, elucidating a novel pathophysiological mechanism of physical activity against TBI.

Iron abnormal deposition is considered as one of the typical characteristics of ferroptosis. It has been widely recognized that iron, an indispensable element, is required for multiple metabolic processes, such as electron transport, tricarboxylic acid (TCA) cycle, and DNA synthesis under normal physiological conditions. Conversely, pathological iron accumulation has proven to be highly associated with poor cognitive outcomes in various neurodegenerative diseases, including TBI [48]. The destruction of vascular integrity and the increased blood–brain barrier permeability induced by TBI leads to the inflow of iron from the circulating blood into the cerebral parenchyma, which results in the severe iron deposition. In addition to promoting the generation of ROS through the Fenton reaction, excess iron also affects the lipid oxidation process and oxygen metabolism by reacting with lipoxygenase (ALOX) or EGLN prolylhydroxylases (PHD). The previous studies provide strong evidence that iron accumulation is closely involved in the pathological process of secondary injury of TBI, and maintaining iron homeostasis is an effective means against ferroptosis triggered by TBI [49]. Therefore, we determined to investigate the mechanisms of abnormal iron metabolism after TBI from three aspects: uptake, storage, and efflux. Iron importer transferrin receptor (Tfr1), identified as the inducer of ferroptosis, can mediate the majority of iron into cells. Iron exporter ferroportin (Fpn), the only known iron exporter until now, functions as extruding iron into the extracellular space [50]. Consistent with previous studies, we found that TBI led to the increase of Tfr1 and the decrease of Fpn [7, 8], which both resulted in an elevated level of intracellular Fe^2+^ in the damaged cortex. In addition, ferritin, the main iron-storage protein, stores nearly 80% of newly imported iron, which plays a vital role in preventing iron-dependent oxidative stress [50]. After passing through pores in the shell, irons enter into the lumen of ferritin and deposit inside the center of catalytic ferroxidase in the form of ferrihydrite [48]. Ferritin heavy chain (Fth), as a main submit of ferritin, is responsible for converting Fe^2+^ to Fe^3+^ [7, 8]. Surprisingly, we found that Fth was upregulated following TBI. And we speculated that the overexpression of Fth may be a self-compensatory response against TBI. Noteworthy, the anti-ferroptosis effects of the excessive Fe^2+^ storage induced by the increase of Fth cannot compete with pro-ferroptosis effects induced by increased inflow and limited outflow of Fe^2+^ triggered by the change of Tfr1 and Fpn after TBI, which eventually leads to the excess cellular iron deposition. In contrast to the previous studies [7], one of our innovative findings is that TBI gives rise to an elevated level of Fe^2+^ in the serum, and its discrepancy may have something to do with different investigative time points. Significantly, treadmill exercise can invert TBI-induced iron overload by increasing the expression of Fth and Fpn and decreasing the level of Tfr1, which provides powerful evidence for the anti-ferroptosis effects of treadmill exercise post brain injury.

The key element of the ferroptosis induction is the inactivation of antioxidant defense, especially the system Xc^−^-glutathione-(GSH)-GPX4 dependent antioxidant pathway [51]. System Xc^−^, a heterodimeric protein complex comprised of SLC3A2 and SLC7A11 (Xct), is a Na^+^-dependent cysteine-glutamate antiporter in the membrane, which is capable of exchanging intracellular glutamate for extracellular cystine at a 1:1 ratio. Noteworthy, system Xc^−^ activation was mainly triggered by the high concentration of intracellular glutamate, which is independent of ATP [52]. Importantly, glutamate excitotoxicity involved in the secondary effects of TBI leads to a significant increase of extracellular glutamate, thereby inactivating the system Xc^−^ and increasing the susceptibility to ferroptosis [7]. After transporting to the cytoplasm, cystine is reduced to cysteine for glutathione (GSH) synthesis. As the rate-limiting enzyme for GSH synthesis, cysteine was remarkably reduced under TBI. GSH, a potent reductant, is a necessary cofactor for glutathione peroxidase 4 (Gpx4). Chen et al. reported that the inactivation of GSH impairs the ability to quench the accumulation of lipid hydroperoxides. Gpx4, a major PLOOH-neutralizing enzyme regarded as the ferroptosis gatekeeper, is capable of reducing phospholipid and cholesterol hydroperoxides to their corresponding alcohols and water [53]. In addition, the depletion of Gpx4 leads to the accumulation of PLOOHs in large concentrations, thereby causing catastrophic and unrepairable membrane injury [54]. In summary, promoting the system Xc^−^-GSH-Gpx4 protects cells from peroxidation damage, and any impairment in the key steps of the Xc^−^-GSH-Gpx4 metabolic pathway ultimately results in ferroptotic cell death. In agreement with previous studies [55], TBI leads to the decrease of Xct, GSH, and Gpx4, and moderate intensity of treadmill exercise effectively reverses the above TBI-induced changes, indicating that treadmill may exert anti-ferroptosis effects partly by restoring impairments of the antioxidant system after TBI.

Unrestrained lipid peroxidation is considered the hallmark of ferroptosis, which can be separated into three phases: initiation, propagation, and termination [50]. In the initiation phase, a co-oxidant (such as OH-) removes a bisallylic hydrogen atom from the methylene carbon, which links the two double bonds of polyunsaturated fatty acyl moieties, and then a carbon-centered phospholipid radical (PL•) is formed. After the reaction with molecular oxygen and ferrous iron, PL• is transformed into lipid peroxy radical (PLOO•), which subsequently reacts with adjacent lipids through a chain of reactions and leads to the formation of phospholipid hydroperoxides (PLOOHs). More importantly, if the initial PLOOH is not cleared rapidly by antioxidant molecules, such as Gpx4 or vitamin E, PLOOHs continue to be converted to lipid-free radicals via that auto-amplifying process, resulting in the propagation of lipid peroxidation. Then, massive PLOOHs are formed and regraded to a myriad of toxic aldehydes, such as 4-hydroxynonenal (4-HNE) and malonaldehyde (MDA), and the level of 4HNE or MDA may indicate the severity of lipid peroxidation. Accumulation of breakdown products finally further affects the membrane integrity and permeability, leading to the rupturing of organelles or membranes and cell death. However, the antioxidant system can terminate the above propagation reactions mainly by providing hydrogen atoms to PLOO•, trapping lipid peroxyl radicals, or reducing lipid hydroperoxide to nontoxic alcohols. During the process of lipid peroxidation, acyl-CoA synthetase long-chain family member 4 (ACSL4), regarded as the key driver of lipid peroxidation, is responsible for ligating the addition of CoA to the long-chain PUFAs such as arachidonic acid and adrenic acid, facilitating the esterification of PUFA to phospholipids [48]. Magtanong et al. reported that the inactivation or loss of ACSL4 makes the long-chain PUFA tails more likely to be converted to short-chain and monounsaturated fatty acyl (MUFA) tails, lowering the propensity to succumb to ferroptosis [56]. In addition, excessive Fe^2+^ generation is crucial for initiating and propagating phospholipid peroxidation. Specifically, Fe^2+^ reacts with hydrogen through the Fenton reaction to generate hydroxyl radical (OH^−^), the most reactive ROS. Then, continuous and excessive production of ROS can cause protein denaturation and destroy cellular lipids, thereby promoting sensitivity to ferroptosis [53]. Consistent with the previous studies [7, 15, 38], we found that TBI induced the increased level of 4HNE, Acsl4, Fe^2+^, and MDA, and treadmill exercise inverted the changes above, suggesting that physical exercise could lessen lipid peroxidation to improve neurological function against ferroptosis-related brain impairments.

Cyclic GMP-AMP (cGAMP) synthase (cGAS), regarded as a double-stranded DNA (dsDNA) sensor, detects equally exogenous and endogenous DNA and then produces the second messenger cyclic guanosine monophosphate-adenosine monophosphate (cGAMP). After the recognition of cGAMP, stimulator of interferon genes (STING) is activated to trigger type I IFN responses and spark the robust inflammatory response [57]. It is known that the accumulation of dead cells, impaired endogenous DNA metabolism, and DNA leakage into the extracellular environment caused by TBI activate cGAS/STING pathway and then facilitate the release of inflammatory cytokines and chemokines. However, growing evidence shows that STING-mediated prolonged and excessive neuroinflammation leads to a toxic microenvironment detrimental to cell viability post TBI [9]. For example, Gamdzyk et al. reported that silencing STING effectively limits the progression of cortical neurodegeneration and improves neurobehavioral outcomes after brain injury [58]. Furthermore, attenuated microglial reactivity and reduced lesion size were also observed in STING^−/−^ mice subjected to TBI, compared to sham group [13]. In addition to the pro-inflammation effects, mounting evidence has proven that STING pathway is closely associated with ferroptosis in the pathological process of pancreatic cancer [59, 60]. Dai et al. reported that ferroptosis induced by a high-iron diet or Gpx4 depletion activates the STING-dependent DNA sensor pathway, facilitating macrophage infiltration and progression of pancreatic cancer, and inhibition of STING pathway mitigates the ferroptosis and suppresses pancreatic tumorigenesis [59]. In addition, Kuang et al. further showed that the accumulation of cysteine protease cathepsin B (CTSB) in the nucleus is the key event for the initiation of ferroptosis, and the activation of STING pathway may be the important mechanism involved in the CTSB-mediated autophagy-dependent ferroptosis [61]. Therefore, it is reasonable to speculate that STING pathway may be involved in the anti-ferroptosis mechanism of physical exercise after TBI. In agreement with our hypothesis, we found that knockdown of STING and physical exercise both significantly ameliorates the excessive iron deposition, lipid peroxidation, and decrease of antioxidant molecules induced by TBI. Moreover, overexpression of STING can largely reverse the above anti-ferroptosis changes of treadmill exercise post TBI. To sum up, we are the first report to demonstrate the vital role of STING-regulated ferroptosis in the neuroprotective effects of treadmill exercise following TBI, potentially indicating a new therapeutic strategy for weakening brain damage.

Growing evidence shows that ferroptosis appears to be the key mechanism of cognitive impairments involved with various neurodegenerative diseases, such as Alzheimer’s disease (AD), Parkinson’s disease (PD), and Huntington’s disease [62]. Bao et al. reported that genetic deletion of Fpn in excitatory neurons of the hippocampus and neocortex by crossing Fpn^fl/fl^ mice with NEX-Cre mice leads to the obvious characteristics of ferroptosis and AD-like hippocampal atrophy and memory damage [63]. Furthermore, the administration of ferroptosis inhibitors can reduce Aβ aggregation-induced memory impairments and neuronal loss, indicating the significant role of ferroptosis in the cognitive dysfunction of AD [63]. In addition, α-syn, the traditionally recognized pathological hallmark of PD, has proven to be functionally linked with the metabolism of ferroptosis, and anti-ferroptosis molecules can delay the onset and progression of PD [64]. More importantly, in a CCI mouse model, administration of ferrostatin-1 to the ventricles remarkably reduces iron deposition and improves long-term cognitive outcomes as measured by the MWM and beam walk tests [8]. Taken together, inhibiting ferroptosis post TBI is considered to be a novel therapeutic strategy to lessen the persistent impaired cognition [65], which is also supported by our findings that inactivation of ferroptosis caused by treadmill exercise effectively reduces the learning and memory deficits, decreases the degree of anxiety, and improves the social novelty. Interestingly, we found a significant loss in social novelty but no impairment in sociability post TBI, which differs from previous studies demonstrating that TBI resulted in obvious deficits in both sociability and social novelty [66]. The investigated time point in our study was 29 days post TBI; however, Ritzel et al. detected the social interaction function at 31 weeks post TBI [66]. Therefore, we speculated that this seemingly inconsistent result is likely because of the difference in the investigated time point post TBI [67]. It is well established that ferroptosis is a major contributor to TBI-caused long-term neurobehavioral disorders [55], and investigating the underlying mechanisms of physical exercise-mediated ferroptosis inactivation after brain injury is necessary. In our study, biochemical and neurobehavioral results demonstrated that the knockdown of STING leads to the inhibition of ferroptosis and a remarkable improvement of cognitive deficits, and physical exercise-induced anti-ferroptosis effects were largely abolished by the overexpression of STING, suggesting that STING pathway may be the critical mechanism of anti-ferroptosis and neural protection effects of treadmill exercise after TBI.

There are several potential limitations deserving attention in our study. Firstly, Sh-STING RNA was used to inhibit the expression of STING, but the data from STING knockout mice is likely to be more persuasive. Secondly, given that the beneficial effects of treadmill exercise involve multiple biologic processes, we cannot rule out the possibility that exercise treatment exerts other neuroprotective effects in facilitating brain function recovery after TBI, apart from inhibiting ferroptosis. Therefore, the multiple interaction mechanisms among ferroptosis and other effects of exercise, such as anti-inflammation and anti-apoptosis, need to be explored further. Furthermore, owing to that only male mice were involved in this study, the gender differences post TBI and the therapeutic effects of exercise in mice of different sex have yet to be investigated. Follow-up studies in female mice are required, aiming to decrease experimental bias caused by gender factors.

## Conclusions

In conclusion, the present study provides sufficient evidence that moderate intensity of treadmill exercise exerts neuroprotective effects through inhibiting ferroptosis, and it might be mediated at least in part by STING pathway. The conclusion is based on three key findings. Firstly, TBI caused the characteristic features of ferroptosis in the chronic stage post TBI, including iron overload, accumulation of lipid peroxides, and imbalanced glutathione metabolism. Secondly, moderate intensity of treadmill exercise and knockdown of STING both reversed the above ferroptosis-related molecular changes, decreased the neurodegeneration level, reduced anxiety and learning and memory deficits, and improved social novelty. Thirdly, overexpression of STING pathway largely reversed the above anti-ferroptosis and cerebral protection effects induced by treadmill exercise post TBI. This study sheds new light on the understanding of the diverse neuroprotective function of physical exercise and provides further insight into investigating the anti-ferroptosis of treadmill exercise post TBI.

## Data Availability

Data will be made available on reasonable request.

## Abbreviations

TBI: Traumatic brain injury
BDNF: Brain-derived neurotrophic factor
I/R: Ischemia-reperfusion
STING: Stimulator of interferon genes
MDA: Malondialdehyde
ROS: Reactive oxygen species
Gpx4: Glutathione peroxidase 4
4HNE: 4-Hydroxynonenal
cGAMP: Cyclic GMP-AMP
GSH: Glutathione
OFT: Open field test
MWM: Morris water maze
FJB: Fluoro-Jade B
Ptgs2: Prostaglandin-endoperoxide synthase 2
Nrf2: Nuclear transcription factor E2-related factor 2
TCA: Tricarboxylic acid
ALOX: Lipoxygenase
PHD: Prolylhydroxylases
Tfr1: Transferrin receptor
Fpn: Ferroportin
NCOA4: Nuclear receptor coactivator 4
UPS: Ubiquitin-proteasome system
Fth: Ferritin heavy chain
GSH: Glutathione
PLOOHs: Phospholipid hydroperoxides
PLOO•: Lipid peroxy radical
PL•: Phospholipid radical
ACSL4: Acyl-CoA synthetase long-chain family member 4
PUFA: Polyunsaturated fatty acid
MUFA: Monounsaturated fatty acyl
dsDNA: Double-stranded DNA
CTSB: Cysteine protease cathepsin B
AD: Alzheimer’s disease
PD: Parkinson’s disease
HD: Huntington’s disease
xCT: A sodium-independent cystine-glutamate antiporter
Lip-1: Liproxstatin-1
